# Integrated RNA Sequencing and QTL Mapping to Identify Candidate Genes from *Oryza rufipogon* Associated with Salt Tolerance at the Seedling Stage

**DOI:** 10.3389/fpls.2017.01427

**Published:** 2017-08-15

**Authors:** Shanshan Wang, Meng Cao, Xin Ma, Weikang Chen, Jie Zhao, Chuanqing Sun, Lubin Tan, Fengxia Liu

**Affiliations:** National Center for Evaluation of Agricultural Wild Plants (Rice), Beijing Key Laboratory of Crop Genetic Improvement, Laboratory of Crop Heterosis and Utilization, MOE, Department of Plant Genetics and Breeding, China Agricultural University Beijing, China

**Keywords:** common wild rice, introgression line, seedling stage, salt tolerance, QTL analysis, RNA-seq

## Abstract

Soil salinity is a common abiotic stress affecting crop productivity. To identify favorable alleles from wild rice (*Oryza rufipogon* Griff.) that enhance salinity tolerance of rice (*O. sativa* L.), a set of introgression lines (ILs) were developed. The ILs were derived from an *O. rufipogon* accession collected from Chaling (Hunan Province, China) as the donor, and a widely grown *O. sativa indica* cultivar 93-11 as the recipient. Through evaluating the salt tolerance of 285 ILs at the seedling stage, a total of 10 quantitative trait loci (QTLs) related to salt tolerance were identified on chromosomes 1, 5, 7 and 9–12, with individual QTLs explaining 2–8% of phenotypic variance. The *O*. *rufipogon*-derived alleles at four QTLs improved salt tolerance in the 93-11 background. At the same time, a salt-tolerant IL, 9L136, was identified and characterized. Compared with the recipient parent 93-11, a total of 1,391 differentially expressed genes (DEGs) were detected specifically in 9L136 between salt stress and normal condition through genome-wide expression analysis. Of these, four DEGs located in the QTL regions carried by 9L136, suggesting that the four genes might be candidates associated with salt tolerance. Both the highly salt-tolerant ILs and the favorable *O. rufipogon*-derived QTLs identified in the present study will provide new genetic resources for improving the resistance of cultivated rice against salinity stress using molecular breeding strategies in the future.

## Introduction

Soil salinity is one of the most common abiotic stresses affecting crop growth and productivity worldwide (Hoang et al., 2016). It is estimated that 20% of the total cultivated and 33% of irrigated agricultural land are affected by salinity (Epstein et al., 1980). Many adaptation and mitigation strategies are required for crops to cope with the impacts of salinity. However, these strategies are cost intensive and time-consuming. Compared with improving saline soil, enhancing resistance of crop plants against salinity stress by breeding and genetic manipulation is an effective and low-cost approach. Rice (*Oryza sativa* L.), one of the most important food crops in the world, has medium sensitivity to salinity stress, and shows symptoms of damage and a dramatic reduction in productivity when the soil soluble salt reaches 0.3% (Akbar et al., 1972). Especially at the seedling stage, salt stress is more likely to cause growth retardation or death of rice, resulting in significant yield reductions (Zeng et al., 2001; Zhao et al., 2014). Therefore, further enhancing salt tolerance at the seedling stage would be helpful to improve environmental adaptation and increase yield in rice.

In the past decades, many QTLs/genes associated with adaptation to salt stress have been identified in rice (Lin et al., 1998, 2004; Koyama et al., 2001; Ren et al., 2005; Huang et al., 2009; Kumar et al., 2015; Liang et al., 2015). One QTL, related to survival time under salt stress at the seedling stage, was detected near the marker locus RG3 on chromosome 5 using a recombinant inbred population, and explained 11.6% of phenotypic variation (Lin et al., 1998). Through evaluating five phenotypic traits associated with salt tolerance, including sodium (Na^+^) and potassium (K^+^) content, a total of 11 QTLs were located on chromosomes 1, 4, 6, and 9, which explained 6.4–19.6% of phenotypic variation (Koyama et al., 2001). In addition, two major QTLs for shoot Na^+^ and K^+^ concentrations, *qSNC-7* and *qSKC-1*, were found on chromosomes 7 and 1, and explained 48.5 and 40.1% of phenotypic variation, respectively (Lin et al., 2004). Using a genome-wide association study for 220 rice accessions, 20 single nucleotide polymorphisms (SNPs) significantly associated with Na^+^/K^+^ ratio, and 44 SNPs with other traits observed under stress conditions were identified at the reproductive stage (Kumar et al., 2015). Additionally, 11 loci containing 22 significant salt tolerance-associated SNPs were identified at the seed germination stage using a similar method (Shi et al., 2017). Through map-based cloning, a salt-tolerance related QTL, *SKC1*, was isolated, which encoded a member of the HKT-type transporters, regulating K^+^/Na^+^ homeostasis under salt stress (Ren et al., 2005). The *DST* gene (*drought and salt tolerance*), encoding a C2H2-type zinc finger transcription factor, was cloned and had a negative effect on drought and salt tolerances through regulating stomatal closure in rice (Huang et al., 2009). In conclusion, salt tolerance is controlled by complex genetic mechanisms in rice, which remain unclear.

Common wild rice (*O. rufipogon* Griff.), the wild progenitor of Asian cultivated rice (*O. sativa* L.), is an important primary gene pool for improving rice. Many favorable genes have been identified and characterized from wild rice (Tian et al., 2006, 2011; Zhang et al., 2006; Tan et al., 2007; Garcia-Oliveira et al., 2009), which can increase rice yield, improve grain quality, and enhance resistance to both biotic and abiotic stresses. Here, to exploit beneficial alleles for salt tolerance from wild rice, we evaluated the salt tolerance of a set of introgression lines (ILs) derived from a cross between an elite *indica* cultivar 93-11 and an *O. rufipogon* accession (Chaling common wild rice) from China, and identified QTLs for salt tolerance. Four candidate genes in QTL regions associated with salt tolerance were identified through integrating RNA sequencing and QTL mapping. The results will aid in utilizing alien alleles to improve tolerance to salt stress in modern rice breeding.

## Materials and Methods

### Plant Materials

A set of ILs was derived from a cross between elite *indica* cultivar, 93-11, and an *O. rufipogon* accession (Chaling common wild rice) from Chaling (Hunan Province, China) based on an advanced backcross (BC_3_) and consecutive selfing strategy (Supplementary Figure S1) as described previously (Ma et al., 2016). A total of 285 ILs were evaluated for salt tolerance and genotype using 142 simple sequence repeat (SSR) markers.

### Phenotypic Evaluation

Seeds of both the recurrent parent 93-11 and 285 ILs were surface-sterilized in 5% sodium hypochlorite for 30 min and washed three times with distilled water, and then germinated at 30°C for 2 days. Finally, uniformly germinated seeds were sown in holes in thin Styrofoam board (with 96 holes and a nylon net bottom), floated on water for 4 days, and then transferred to float on Yoshida’s culture solution (Yoshida et al., 1976; Tian et al., 2011). About 14 days after germination, 10 normal seedlings of each line and the recurrent parent 93-11 with two and a half leaves were transferred to culture solution containing 125 mM NaCl and full-strength Yoshida’s solution. Both the survival rate and salt tolerance score (STS) of each line were measured after 9 days of salt treatment and 7 days of recovery. The survival rate was the percentage of living seedlings in the total number of seedlings. The STS was divided into five grades as described by Gregorio and Senadhira (1993) and Tian et al. (2011) with some modification (Supplementary Figure S2): Grade 0 included seedlings that died or were close to death; Grade 1 had significantly inhibited seedling growth and a survival rate of leaves of less than 20%; Grade 2 had some inhibition of seedling growth, plants had three leaves, and a survival rate of leaves of 20–40%; Grade 3 included seedlings with normal growth, plants that reached the four-leaf stage, and a survival rate of leaves of 40–60%; Grade 4 was seedling growth close to normal and a survival rate of leaves of 60–80%; and Grade 5 was normal seedling growth, with only the leaf tips of leaves being dry, and a survival rate of leaves of 80–100%.

### DNA Extraction and SSR Analysis

Using the cetyltrimethyl ammonium bromide method (Rogers and Bendich, 1988), DNA of fresh leaves was extracted after grinding in liquid nitrogen. Primers to amplify SSR sequences were synthesized based on sequence information (McCouch et al., 2002). PCR amplification, polyacrylamide denaturing gel electrophoresis, and silver-staining were performed as described previously (Panaud et al., 1996).

### Genotyping

Based on the genotypic analysis of 285 ILs using 142 polymorphic SSR markers, QTLs for salt tolerance were determined using single-point analysis in Map Manager QTXb17 (Manly et al., 2001). The statistical threshold for single-point analysis was *P* < 0.01 and LOD ≥ 2. The proportion of observed phenotypic variance explained by a QTL was estimated by the coefficient of determination (*R*^2^) from the corresponding linear model fitting.

### Measurement of Physiological Characteristics

Six physiological characteristics associated with abiotic stress were measured as described previously (Yemm and Willis, 1954; Beauchamp and Fridovich, 1971; Dhindsa et al., 1981; Nakno and Asada, 1981; Ie-sung et al., 2003): contents of proline and soluble sugars, and activities of malondialdehyde, superoxide dismutase, catalase, and peroxidase. Both 93-11 and a highly salt-tolerant IL 9L136 were grown to the two and a half leaf-stage, and then transferred to culture solution containing 125 mM NaCl for the salt treatment. The seedlings were harvested quickly at different time points (0, 1, 2, 3, and 4 days after salt treatment) for measurement of physiological characteristics. Each experiment was replicated three times.

### RNA-seq Analysis

Total RNA was isolated from roots of the recurrent parent 93-11 and IL 9L136 under salt treatment (1 h after treatment, ST) and control (0 h, CK) using TRizol (Invitrogen, Carlsbad, CA, United States) and purified using a Qiagen RNeasy kit (Qiagen, Germany). Each sample had three biological replicates. The purified RNA was used to construct the cDNA library using a NEBNext Ultra RNA Library Prep kit. The 12 libraries (including root samples from 9L136 and 93-11 before and 1 h after application of salt stress, and each sample had three biological repeats, *n* = 3) were subsequently sequenced using an Illumina HiSeq2500 Sequencer. After removing adaptor sequences and low-quality reads, the high-quality paired-end reads were mapped to the Nipponbare reference genome (MSU Rice Genome Annotation Project Release 7) using the spliced read mapper TopHat version 2.0.12 (Kim et al., 2013). Principal component analysis (PCA) was performed using the prcomp function in R software with default settings to interpret the relatedness among all replicas in each genotype (R Development Core Team, 2012). Significant differentially expressed genes (DEGs) were determined based on a threshold of twofold expression change and false discovery rate (FDR) < 0.05 using Cuffdiff, one module of Cufflinks (Trapnell et al., 2012).

### Validation of RNA-Seq Data by Quantitative Real-Time RT-PCR (qRT-PCR)

RNA samples were reverse transcribed into cDNA using a SuperScript III RT kit (Invitrogen, Carlsbad, CA, United States; Cat. No. 18080-044). The cDNA samples were subjected to qRT-PCR quantification with three biological replicates and performed with SYBR Green Master Mix (Applied Biosystems, Foster City, CA, United States; PN 4309155) according to the product manual on a CFX96 Real Time System (Bio-Rad, Hercules, CA, United States). Reference genes and specific gene primers are listed in Supplementary Table S1. The relative quantification method was used to evaluate the quantitative variation between the replicates examined.

## Results

### Evaluation of Salt Tolerance of Wild Rice Introgression Lines at the Seedling Stage

**FIGURE 1:**
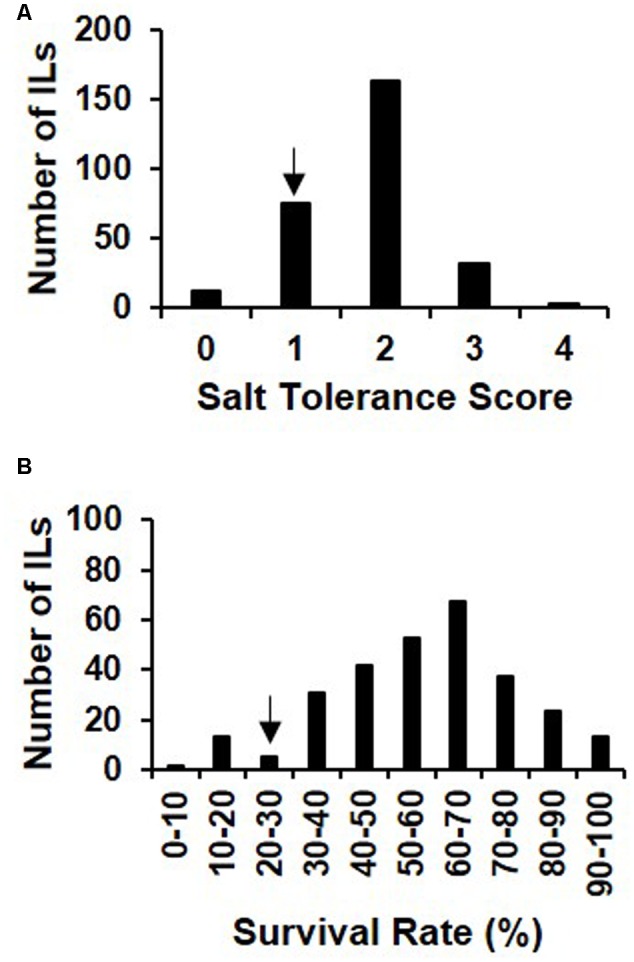
Frequency distribution of salt tolerance score **(A)** of seedlings and survival rate **(B)** in introgression lines under 125 mM NaCl treatment for 9 days and recovery for 7 days. The phenotype of the recurrent parent, 93-11, is indicated by arrows.

To identify *O. rufipogon*-derived favorable alleles associated with salt tolerance, we investigated both STS and survival rate of 285 ILs and the recurrent parent 93-11 under 125 mM NaCl treatment. Phenotypic measurement showed that the frequency distributions of the two traits in the IL population accorded with the normal distribution, and both STS and the survival rate of the recurrent parent 93-11 (1.07 and 43%) were lower than the means for ILs (1.81 and 54%) at the seedling stage (**Figure 1** and **Table 1**). The results indicated that the recurrent parent 93-11 was more susceptible to salt stress, and that the *O. rufipogon* genome might harbor beneficial alleles associated with enhanced salt tolerance in the cultivated rice background.

**Table 1:** Observations of salt tolerance in the recipient parent 93-11 and introgression lines at the seedling stage.

Traits	93-11	Introgression lines				
		Average	Range	Coefficient of variation	Skewness	Kurtosis
STS	1.07	1.81 ± 0.67	0.07–3.82	0.37	–0.07	0.21
Survival rate	43%	54 ± 16%	7–100%	0.3	–0.43	0.33

### QTL Analysis for Salt Tolerance at the Seedling Stage

**FIGURE 2:**
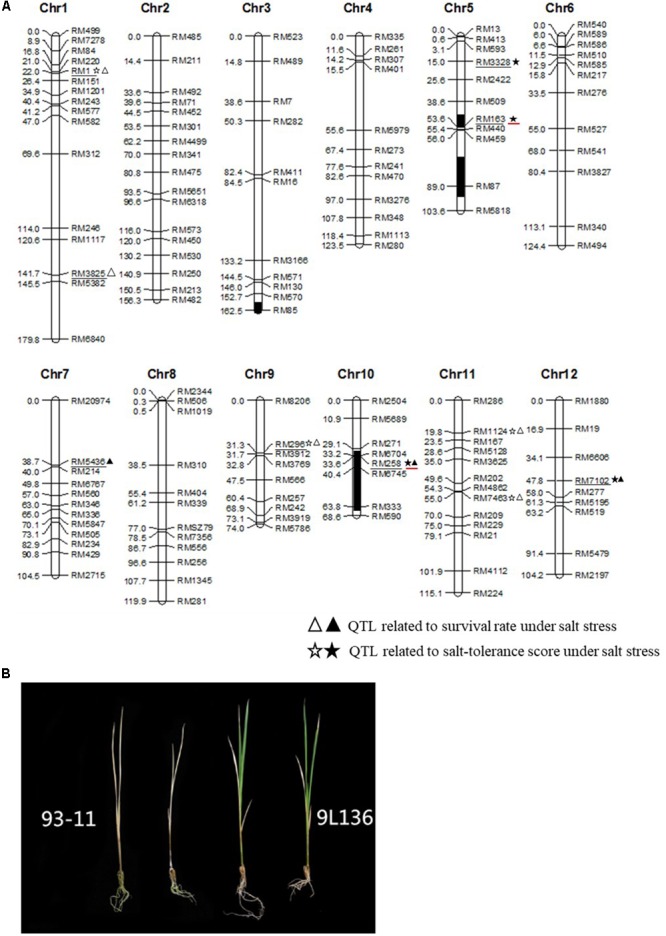
Chromosomal location of salt tolerance-related QTLs **(A)** and the 9L136 and 93-11 phenotypes treated with 125 mM NaCl for 9 days **(B)**. Symbols on the right of chromosomes indicate the positions of putative QTLs for salt tolerance-related traits. The closed symbols indicate that the *O. rufipogon*-derived alleles conferred a positive effect on salt tolerance-related traits, and the open symbols indicate a negative effect. The markers related to QTLs for salt tolerance are underlined with black. In the graphical genotypes, the black regions indicate homozygous regions from Chaling common wild rice in 9L136 and the white regions indicate homozygous regions for recurrent parent 93-11. The QTLs enhancing salinity tolerance and located on the introgressed region in 9L136 are underlined with red.

Based on the genotypic analysis of 285 ILs using 142 polymorphic SSR markers, a total of 1358 chromosomal segments from *O. rufipogon* were detected, including 1082 homozygous and 276 heterozygous segments (Supplementary Table S2). Using single-point analysis, a total of 10 QTLs associated with salt response were identified (**Figure 2** and **Table 2**). They were located on chromosomes 1, 5, 7 and 9–12, and each QTL explained 2–8% of the phenotypic variation (**Figure 2** and **Table 2**). Additionally, the *O. rufipogon*-derived alleles at four loci (40% of QTLs) improved salt tolerance. Among these QTLs, six located on chromosomes 1 and 9–12 were detected using STS and survival rate as indexes. For example, *qST10* was located near the SSR marker RM258 on chromosome 10, and *qST12* near SSR marker RM7102 on chromosome 12. Moreover, alleles from *O. rufipogon* at the two QTLs could improve salt tolerance at the seedling stage in the 93-11 background. However, the other four QTLs (*qST5-1, qST5-2, qST1-2* and *qST7*) were only detected by one index.

**Table 2:** QTLs for salt tolerance at the seedling stage identified using *O. rufipogon* introgression lines.

Traits	Chr.	QTL	Marker	LOD	PV (%)	*P*-value	Add.
STS	1	*qST1-1*	RM1	3	4	0.001	–0.3
	5	*qST5-1*	RM3328	2	8	0.003	0.64
	5	*qST5-2*	RM163	2	8	0.006	0.62
	9	*qST9*	RM296	3	4	0	–0.26
	10	*qST10*	RM258	2	4	0.001	0.42
	11	*qST11-1*	RM1124	3	5	0.001	–0.69
	11	*qST11-2*	RM7463	3	5	0.001	–0.45
	12	*qST12*	RM7102	2	4	0.001	0.43
Survival rate	1	*qST1-1*	RM1	3	5	0	–0.08
	1	*qST1-2*	RM3825	2	2	0.009	–0.04
	7	*qST7*	RM5436	2	4	0.007	–0.11
	9	*qST9*	RM296	3	5	0	–0.06
	10	*qST10*	RM258	2	4	0.002	0.09
	11	*qST11-1*	RM1124	2	5	0.001	–0.12
	11	*qST11-2*	RM7463	2	4	0.001	–0.07
	12	*qST12*	RM7102	2	4	0.007	0.12

*Chr., chromosome; PV, phenotypic variation; Add., additive effect.*

### Analysis of Salt-Tolerant Introgression Lines

To select the genetic materials for further breeding salt-tolerant rice varieties and cloning salt tolerance-related genes, we performed six independent-experiment evaluations and identified four ILs (9L19, 9L46, 9L136, and 9L201), which showed stable salt tolerance in multiple experiments, and had significantly higher STS and survival rates than those of the recurrent parent 93-11 (**Table 3**). Comparison of introgression segments with chromosomal location of QTLs carried by ILs showed that the four lines harbored two positive-effect QTLs for salt tolerance from *O. rufipogon* (**Table 3** and Supplementary Figure S3). Notably, the highly salt-tolerant IL 9L136 carrying two salt tolerance-related QTLs (*qST5-2* and *qST10*) (**Figure 2**), had the highest STS and survival rate among the four ILs, and its average survival rate was 84%, which increased by 95.3% compared with 93-11 (**Table 3**). Therefore, 9L136 was chosen for further characterization, as a highly salt-tolerant IL.

**Table 3:** QTLs included in the introgressed segments carried by four selected salt-tolerant introgression lines.

Number	Salt-tolerance	Survival	QTLs
of ILs	score	rate	included
9L19	2.80 ± 1.83	63 ± 40%	*qST5-1, qST5-2*
9L46	3.11 ± 1.23	76 ± 25%	*qST5-1, qST5-2*
9L136	3.67 ± 0.69	84 ± 12%	*qST5-2, qST10*
9L201	2.48 ± 1.31	60 ± 25%	*qST5-1, qST1-2*
93-11	1.07 ± 0.53	43 ± 31%	

### Physiological Characteristics of the Salt-Tolerant Line 9L136

**FIGURE 3:**
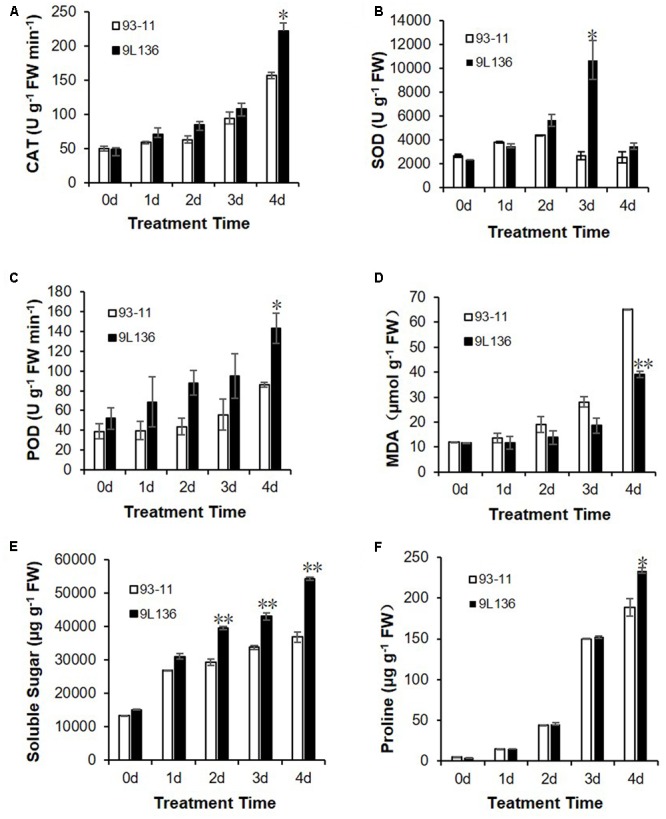
Comparison of physiological characteristics between salt-tolerant line 9L136 and recurrent parent 93-11 under salt stress conditions (125 mM NaCl). Physiological characteristics included the activities of catalase (CAT) **(A)**, superoxide dismutase (SOD) **(B)**, peroxidase (POD) **(C)**, and malondialdehyde (MDA) **(D)**, and the contents of soluble sugars **(E)** and proline **(F)**. Data are means ± standard deviation with three replicates. Asterisks represent significant differences between 93-11 and 9L136 using Student’s *t-*test: ^∗^*P* < 0.05 and ^∗∗^*P* < 0.01.

Generally, salt stress is accompanied by osmotic stress, leading to reactive oxygen species damage and osmolyte accumulation (Abraham et al., 2003; Mittler et al., 2004; Miller et al., 2010; Farmer and Mueller, 2013). To investigate the physiological basis for the improved stress tolerance of 9L136, we measured the contents of proline and soluble sugar and activities of malondialdehyde, superoxide dismutase, catalase, and peroxidase in 9L136 and 93-11 under normal and salt-stress conditions. The activities of superoxide dismutase, catalase, and peroxidase were all significantly higher in 9L136 than 93-11 under salt stress (*P* < 0.05, **Figures 3A–C**), and 9L136 had a distinctly lower malondialdehyde content in response to 4 days of salt treatment compared with 93-11 (*P* < 0.01, **Figure 3D**). We also investigated the contents of soluble sugars, a class of compatible osmolytes, and observed that this was significantly higher in 9L136 than in 93-11 under salt treatment for 2, 3, and 4 days (**Figure 3E**). Additionally, the proline content in 9L136 was significantly higher than that in 93-11 under salt stress (**Figure 3F**). Overall, the results implied that 9L136 did not suffer from oxidative stress as much as the recurrent parent 93-11 during salt stress, suggesting that favorable alleles from wild rice carried by 9L136 might improve the salt tolerance of rice in the 93-11 background.

### Identification of Salt Tolerance-Related Candidate Genes in Wild Rice

To explore candidate genes related to salt tolerance, RNA-seq analysis was performed using RNA extracted from roots of 93-11 and 9L136 after 0 (CK) and 1 h (ST) treatment with 125 mM NaCl. A total of 692 million clean reads (FPKM of all replicas cufflinks showed in Supplementary Table S3) were obtained from 12 libraries, and about 80% of the reads could be mapped to predict gene regions. Sequence read information is summarized in Supplementary Table S4. Both Pearson’s correlation and PCA showed a high correlation among the replicas in each genotype (Supplementary Figure S4, S5 and Table S5). We also validated the expression levels of 15 randomly selected genes using qRT-PCR, which gave similar results to those of RNA-seq analysis – suggesting that RNA-seq data were reliable (Supplementary Figure S6).

**FIGURE 4:**
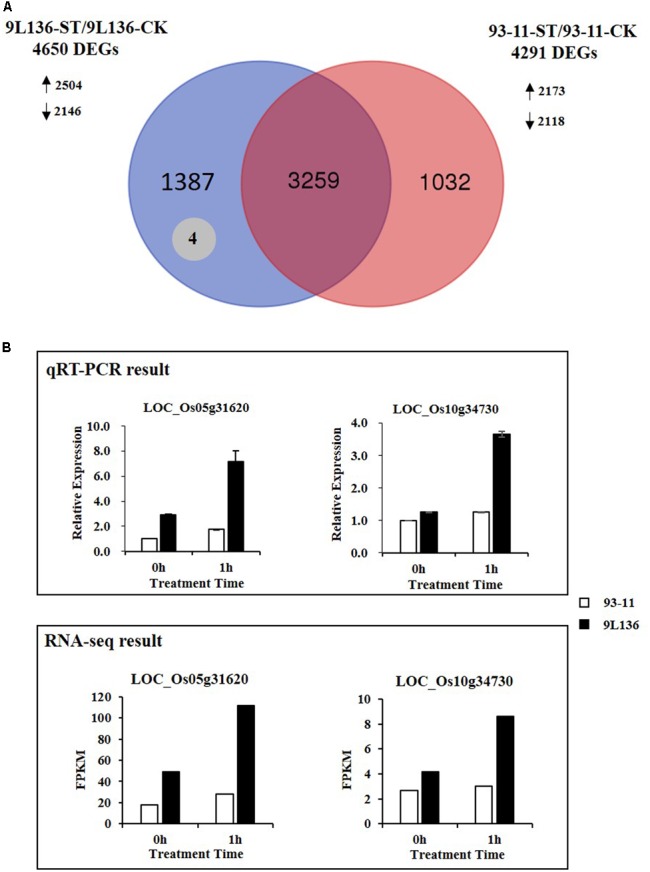
Identification of candidate genes related to salt tolerance from wild rice. **(A)** Venn diagram analysis for RNA-seq data showing differentially expressed genes between 9L136-ST/9L136-CK and 93-11-ST/93-11-CK. There were 1,391 genes showed differentially expressed only in 9L136-ST/9L136-CK but not in 93-11-ST/93-11-CK **(A)**, suggesting these genes should be specifically induced in introgression line 9L136. Among the 1,391 genes, four were co-localized with QTLs for salt tolerance (shown in gray circle). Arrows indicated the up-regulated (↑) and down-regulated (↓) genes. **(B)** Expression profiles of LOC_Os05g31620 and LOC_Os10g34730 detected by qRT-PCR and RNA-seq, respectively.

Global expression analysis showed that a total of 4,650 DEGs, including 2,504 up-regulated and 2,146 down-regulated genes, were detected in IL 9L136 between salt stress (9L136-ST) and normal condition (9L136-CK); and a total of 4,291 DEGs, including 2,173 up-regulated and 2,118 down-regulated genes, were detected in the recipient parent 93-11 between salt stress (93-11-ST) and normal condition (93-11-CK) (**Figure 4A**). Further comparison of DEGs between 9L136-ST/9L136-CK and 93-11-ST/93-11-CK found that 1,391 genes showed differentially expressed only in 9L136-ST/9L136-CK but not in 93-11-ST/93-11-CK (**Figure 4A**), suggesting that these genes should be specifically induced in IL 9L136 under salt stress.

Additionally, based on the gene annotation information of the rice reference genome (the TIGR Rice Genome Annotation Database), chromosomal regions of two QTLs (*qST5-2* and *qST10*) carried by 9L136 harbored a total of 209 genes. To narrow down the candidate genes for salt tolerance, we further compared the chromosomal location between the 1,391 DEGs and QTL regions, and found that four genes, including *acetyltransferase* (LOC_Os05g31254), *calmodulin-related calcium sensor protein* (*OsCML15*, LOC_Os05g31620), *LRP1* (LOC_Os05g32070), and *GRAM domain containing protein* (GEM, LOC_Os10g34730), were co-localized with the QTLs (*qST5-2* and *qST10*) (Supplementary Table S6). Of the four genes, two ones (LOC_Os05g31620 and LOC_Os10g34730) were not only specifically induced by salt stress in 9L136 but also showed higher expression level in 9L136-ST compared with 93-11-ST (**Figure 4B**). Taken together, four candidate genes associated with salt tolerance at the seedling stage were identified through the strategy of combining QTL mapping and genome-wide expression analysis.

## Discussion

### Comparison of Salt Tolerance-Related QTLs in Different Populations

In previous studies, many QTLs related to salt tolerance were identified and located on 12 rice chromosomes, with most of them identified in cultivated rice. There are few reports of QTLs for salt tolerance in common wild rice. A total of 15 QTLs for salt tolerance were identified on chromosomes 1, 2, 3, 6, 7, 9, and 10 using a set of ILs derived from Yuanjiang common wild rice, and the wild rice derived alleles at 13 QTLs (86.7%) could enhance salt tolerance in the Teqing background (Tian et al., 2011). In our study, we used 285 ILs derived from a cross between elite *indica* variety 93-11 and an accession of Chaling common wild rice to identify 10 QTLs for salt tolerance on chromosomes 1, 5, 7, and 9–12. Comparison of salt tolerance-related QTLs in this study with those reported by Tian et al. (2011) showed that QTL *qST7* mapped near RM5436 on chromosome 7 shared a similar chromosomal region with three QTLs (*qRRW7, qRSW7*, and *qRTW7*); and *qST10*, detected on chromosome 10 near RM258, was located on a similar region with a QTL cluster (*qRRW10, qRSW10*, and *qRTW10*) reported by Tian et al. (2011). Interestingly, the *O. rufipogon*-derived alleles at these QTLs/QTL cluster improved salt tolerance in the cultivated rice background.

We also compared the QTLs in this study with those detected in *O. sativa* populations. The results showed that *qST5-1* near RM3328 on chromosome 5 was in a similar location to *qDLRa5-2* and *QSst5a* (Qian et al., 2009; Liang et al., 2015), and *qST5-2* was located on a similar location to *qDSRs5-1, QDss5, QSf5*, and *QGW5* (Chen et al., 2008; Liang et al., 2015), further confirming the reliability of the salt stress-related QTLs detected in the present study. Moreover, the results indicated that the stable QTLs might be useful for rice breeding using marker-assisted selection and so accelerate the development of salt-tolerant rice varieties.

### Possible Physiological and Molecular Mechanisms Underlying the Salt-Stress Response

Through both cellular and physiological changes, crop plants can respond and adapt to abiotic stresses, thus enabling them to survive (Huang et al., 2012; Kumar et al., 2013; Hoang et al., 2016). Under salt stress, the cell membrane is damaged due to salt damage, resulting in malondialdehyde production. Malondialdehyde is an indicator of reactive oxygen species level and the extent of damage to the membrane, and many free radicals are generated by this damage (Farmer and Mueller, 2013). Peroxidase and catalase are protective enzymes activated after superoxide dismutase, and further remove peroxide and hydrogen peroxide (Apel and Hirt, 2004; Jaleel et al., 2009). In our study, activity of protective enzymes catalase, superoxide dismutase, and peroxidase in 9L136 under salt-stress conditions were significantly higher than in the recurrent parent 93-11. Moreover, the superoxide dismutase increased significantly at 3 days of salt treatment and other substances (peroxidase, catalase, and malondialdehyde) had significant levels at 4 days of salt treatment. This indicated that the superoxide dismutase may remove the initially produced O_2_^–^ and H_2_O_2_, and then peroxidase and catalase further removed or decomposed H_2_O_2_ (Pan et al., 2006), leading to lower reactive oxygen species levels in 9L136 than in 93-11 during salt stress. In addition, the contents of proline and soluble sugars in 9L136 under salt-stress conditions were significantly increased compared with 93-11. The results implied that the cell membrane in 9L136 was protected through increased activities of enzymes that scavenge reactive oxygen species, and more proline and soluble sugars under salt stress conditions, leading to improved salt tolerance.

### Implication of Genes for Salt-Stress Tolerance

Through the platform combining QTL mapping and RNA-seq analysis, four candidate genes were co-localized with QTLs for salt tolerance, including *acetyltransferase* (LOC_Os05g31254), *calmodulin-related calcium sensor protein* (*OsCML15*, LOC_Os05g31620), *LRP1* (LOC_Os05g32070), and *GRAM domain containing protein* (GEM, LOC_Os10g34730). Notably, both LOC_Os05g31620 (*calmodulin-related calcium sensor protein, OsCML15*) and LOC_Os10g34730 (*GRAM domain containing protein*, GEM) had higher expression level in 9L136-ST than in 93-11-ST. There are many GRAM domain-containing protein genes in rice, but only one GRAM domain-containing protein has been reported in rice and may function in plant development (Shu et al., 2005). In *Arabidopsis*, VAD1, a novel GRAM domain-containing protein was first characterized and its expression was in response to a pathogen-infection-dependent salicylic acid pathway (Lorrain et al., 2004). The gene LOC_Os10g34730 may function in stress defense and result in salt tolerance of rice by control of their expression.

We are most interested in *OsCML15*, the calmodulin-related calcium sensor protein gene located in the QTL region. In *Arabidopsis*, several *CML*s have dramatic expression changes under various biotic and abiotic stimuli. *CML10* can modulate stress responses in *Arabidopsis* by regulating ascorbic acid production (Cho et al., 2016). *CML24* may enable responses to abscisic acid, daylength, and various salt stresses (Delk et al., 2005). In young *Arabidopsis* seedlings, *AtCML9* expression is rapidly induced by abiotic stress and abscisic acid, and mutation analysis showed that it plays essential roles in modulating responses to salt stress and abscisic acid (Magnan et al., 2008). Additionally, in rice, a novel rice calmodulin-like gene, *OsMSR2*, was identified, which can enhance drought and salt tolerance and increase abscisic acid sensitivity in *Arabidopsis* (Xu et al., 2011). Consequently, further study of *OsCML15* will be valuable for determining salt-stress mechanisms and in saline-resistance breeding.

## Availability Of Supporting Data

The original RNA-seq data has been uploaded to NCBI (https://www.ncbi.nlm.nih.gov/geo/), and the GEO accession number is GSE101734.

## Author Contributions

Manuscript draft: SW, FL, XM, LT, and CS. Experiments: SW, MC, JZ, and WC. Analyzing data: XM. All authors read and approved the final manuscript.

## Conflict of Interest Statement

The authors declare that the research was conducted in the absence of any commercial or financial relationships that could be construed as a potential conflict of interest. The reviewer MJ and handling Editor declared their shared affiliation, and the handling Editor states that the process nevertheless met the standards of a fair and objective review.
