# Supplementary material for: Integrated RNA Sequencing and QTL Mapping to Identify Candidate Genes from Oryza rufipogon Associated with Salt Tolerance at the Seedling Stage
Source: Front Plant Sci. 2017 Aug 15;8:1427. doi: 10.3389/fpls.2017.01427 (PMC5559499; doi:10.3389/fpls.2017.01427)
Supplement: Supplementary file 7 [file Presentation_1.PDF]

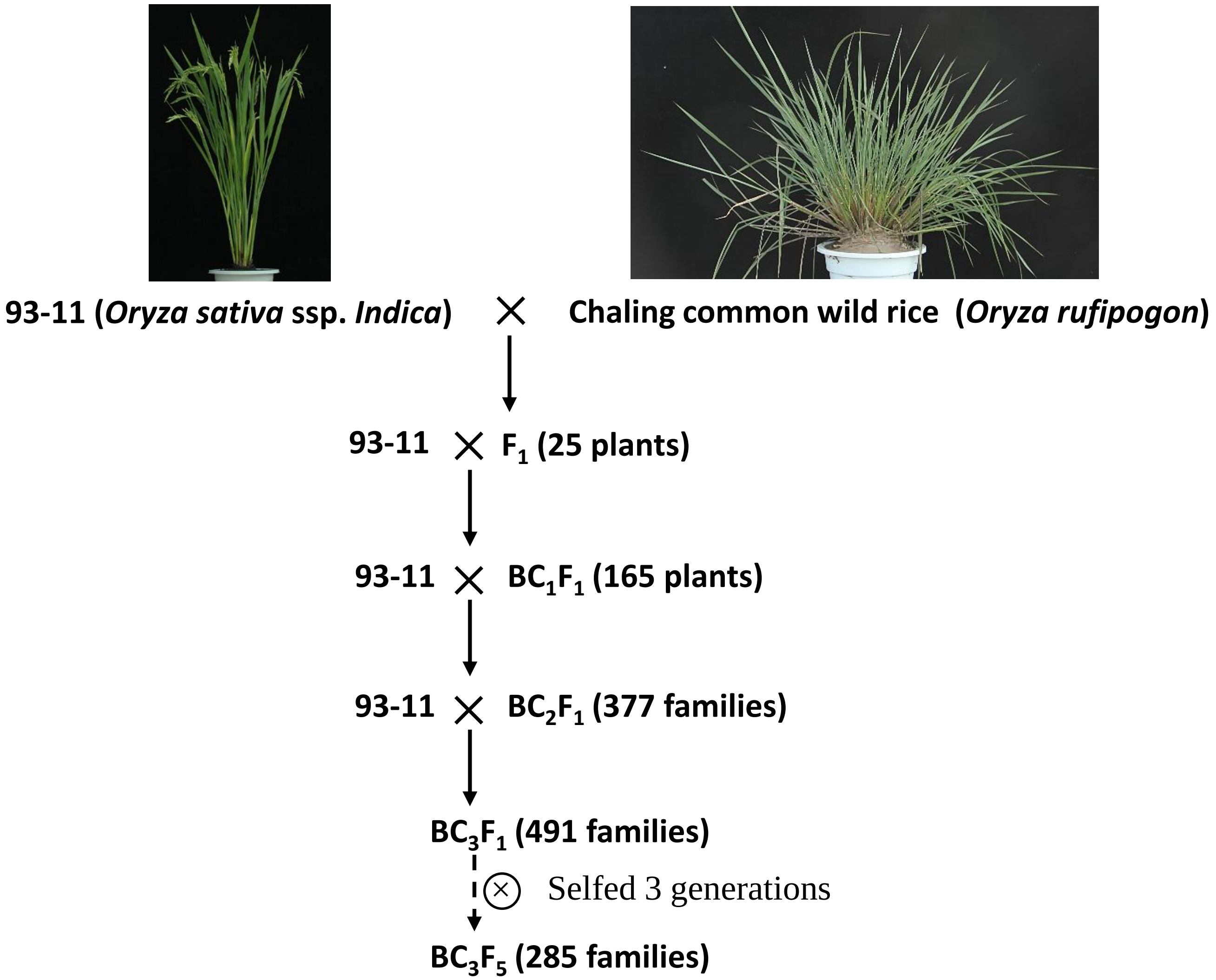

Figure S1 Development of wild rice introgression line population.

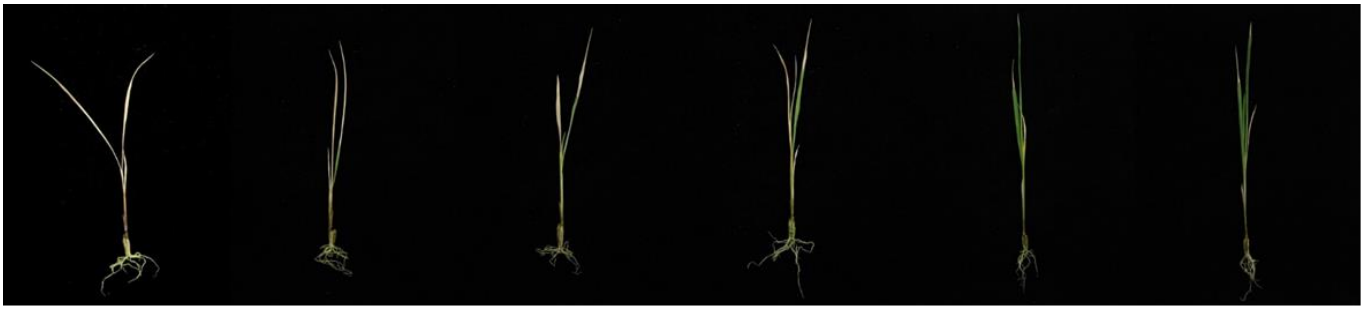

Figure S2 Salt-tolerance phenotypes of introgression lines after 125 mM NaCl treatment for 9 d in the seedling stage.

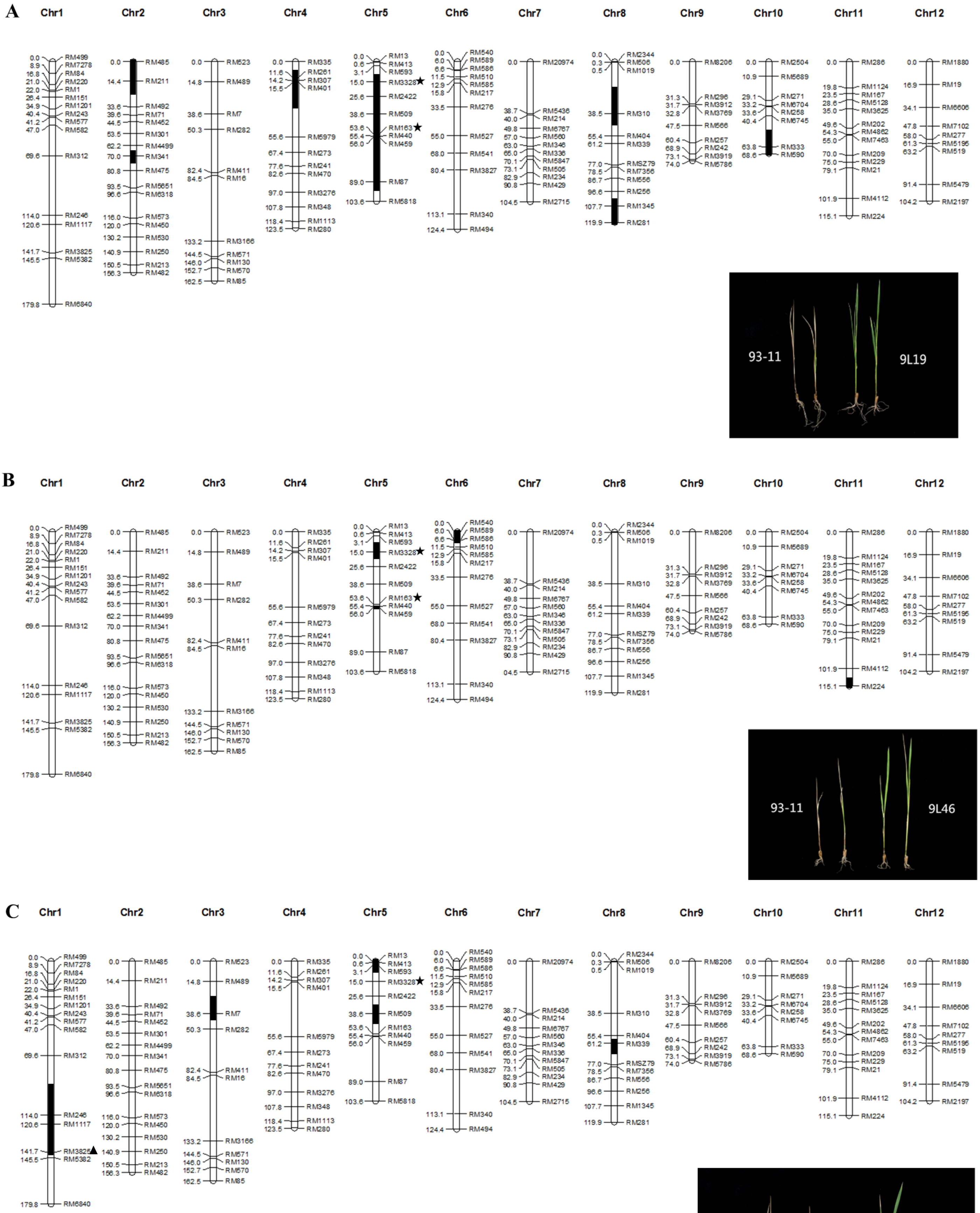

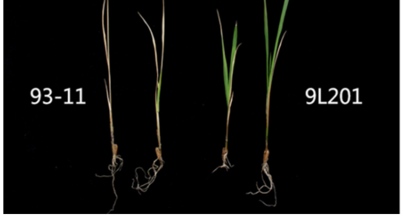

Figure S3 The graphical genotype and phenotype (treated with 125 mM NaCl for 9 d) of salt-tolerant lines (9L19, 9L46, and 9L201) and 93-11. In the graphical genotype, the black regions indicate homozygous regions for common wild rice and the white regions indicate homozygous regions for recurrent parent 93-11.

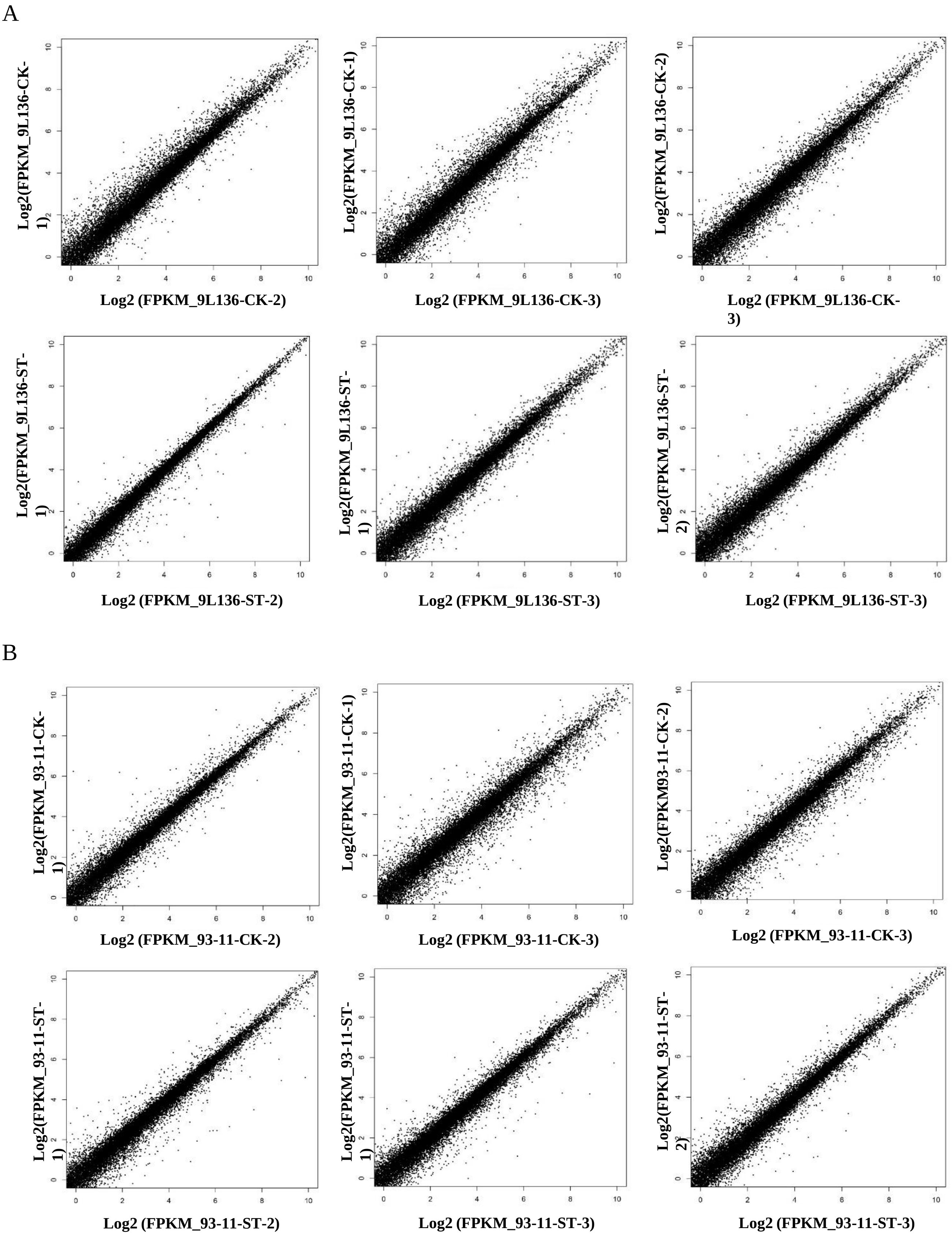

Figure S4 Pearson's correlation coefficients among biological replicates. (A) Correlations among three biological replicates of 9L136-CK (9L136-CK-1, 9L136-CK-2, and 9L136-CK-3) and 9L136-ST (9L136-ST-1, 9L136-ST-2, and 9L136-ST-3). (B) Correlations among three biological replicates of 93-11-CK (93-11-CK-1, 93-11-CK-2, and 93-11-CK-3) and 93-11-ST (93-11-ST-1, 93-11-ST-2, and 93-11-ST-3).

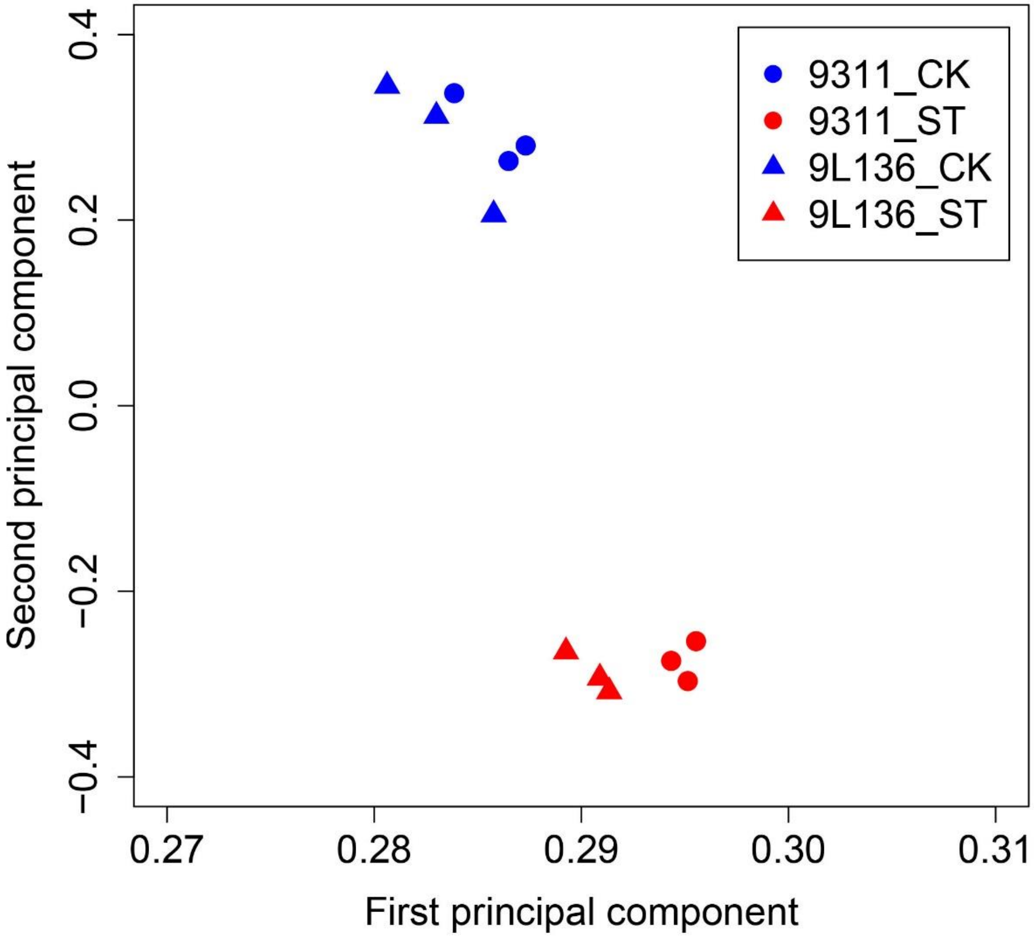

Figure S5 Principal component analysis (PCA) for all replicas in each genotype.

A

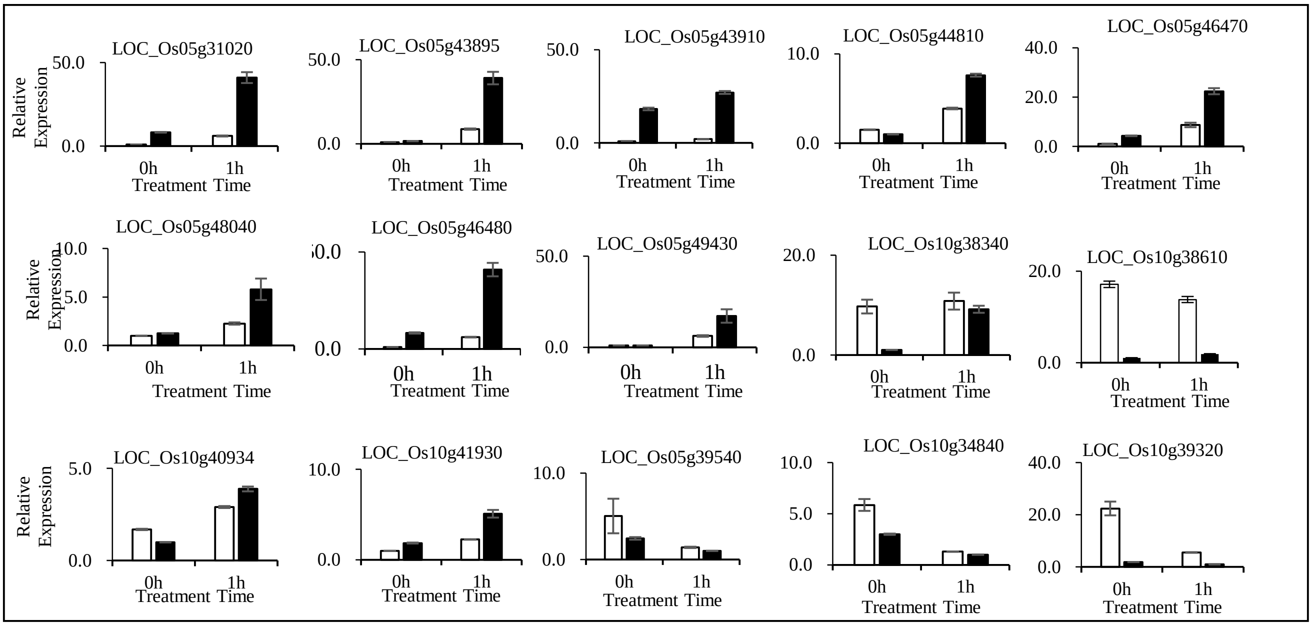

B

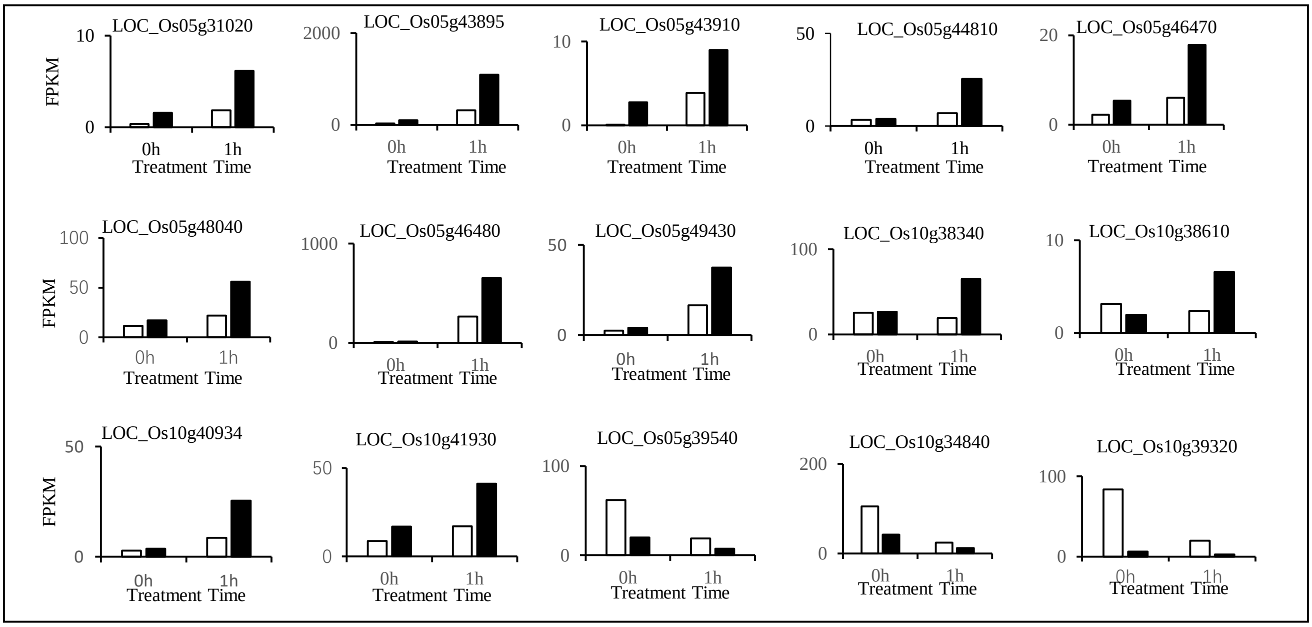

Figure S6 Validation of expression profile for 15 randomly selected genes using qRT-PCR. A, qRT-PCR result for 15 genes; B, RNA-seq result for 15 genes.
